# Nanobubble Mediated Gene Delivery in Conjunction With a Hand-Held Ultrasound Scanner

**DOI:** 10.3389/fphar.2020.00363

**Published:** 2020-04-01

**Authors:** Hiroshi Kida, Koyo Nishimura, Koki Ogawa, Akiko Watanabe, Loreto B. Feril, Yutaka Irie, Hitomi Endo, Shigeru Kawakami, Katsuro Tachibana

**Affiliations:** ^1^Department of Anatomy, Fukuoka University School of Medicine, Fukuoka, Japan; ^2^Department of Pharmaceutical Informatics, Graduate School of Biomedical Sciences, Nagasaki University, Nagasaki, Japan

**Keywords:** gene transfection, sonoporation, ultrasound, nanobubble, ultrasound diagnostic device

## Abstract

Recent research has revealed that nanobubbles (NBs) can be an effective tool for gene transfection in conjunction with therapeutic ultrasound (US). However, an approach to apply commercially available hand-held diagnostic US scanners for this purpose has not been evaluated as of now. In the present study, we first compared *in vitro*, the efficiency of gene transfer (pCMV-Luciferase) with lipid-based and albumin-based NBs irradiated by therapeutic US (1MHz, 5.0 W/cm^2^) in oral squamous carcinoma cell line HSC-2. Secondly, we similarly examined if gene transfer in mice is possible using a clinical hand-held US scanner (2.3MHz, MI 1.0). Results showed that lipid-based NBs induced more gene transfection compared to albumin-based NBs, *in vitro*. Furthermore, significant gene transfer was also obtained in mice liver with lipid-based NBs. Sub-micro sized bubbles proved to be a powerful gene transfer reagent in combination with conventional hand-held ultrasonic diagnostic device.

## Introduction

For the past two decades, there has been much research on ultrasound (US)-mediated drug delivery. Microbubbles (MBs) are considered to play a major role in increasing permeabilization of various drugs through the cell membrane and into the cytoplasm (Tachibana et al., 1999). Recent experiments using high speed video cameras under optic microscopes have shown that oscillation of MBs during US irradiation actually disrupts the cell membrane to form transient pores (Kudo et al., 2009; Nejad et al., 2016; Song et al., 2019). This phenomenon, frequently referred as sonoporation (Lammertink et al., 2015), induces increase in cell uptake of administrated drugs, molecules, and in some cases plasmid DNA (Sennoga et al., 2017). Consequently, the combination of MB and US has been proposed as a novel therapeutic approach to deliver functional genetic molecules such as pDNA, siRNA, antisense oligonucleotides to cancer cells, normal tissues, and various organs for the purpose of preventing or treating diseases.

Previous studies have suggested that altering the MB shell compositions is of great importance for the purpose of increasing the efficacy of drug delivery (Dicker et al., 2013; Abenojar et al., 2019). MB shells that have targeting ligands are especially attractive from the viewpoint of concentrating functional genes to a specific site for gene therapy. Furthermore, such shell compositions as albumin and lipids can serve as a versatile carrier for various types of drugs (Paefgen et al., 2015). Drug incorporated MBs can be destructed and payload released at the site of interest by externally applied therapeutic US (Cochran et al., 2011; Zhu et al., 2016). A considerable amount of gene therapy related literature exists detailing application of the above technique to treat various tissues and organs, including skeletal muscles (Taniyama et al., 2002a; Li et al., 2003), blood vessels (Taniyama et al., 2002b), cornea (Sonoda et al., 2006), and the brain (Negishi et al., 2015).

Generally, the MB used for drug delivery is between 1 and 10 µm in diameter. These sizes which are similar to red blood cells, limit MB extravasation from the bloodstream into the extravascular space in tumors and normal tissues. This becomes a major obstacle when taking into account the necessity to have the therapeutic gene and MB at close proximity to the target cell when irradiating US to a localized site. On the other hand, sub-micron sized NBs (recently officially termed as ultra-fine bubbles) have a potential advantage over MBs due to the fact that having a diameter in the nanoscale makes possible for them to extravasate through the vascular wall structure (Wu et al., 2013). Furthermore, NBs injected into the bloodstream can more easily accumulate in tumor vasculature due to the enhanced permeability and retention (EPR) effect.

Recent advances in acoustic technology have led to the miniaturization of diagnostic US devices to the size of laptop computers and mobile phones. These so called hand-held US scanners are used for bedside physical examination and have become a common place modality (Lapostolle et al., 2006). Although commercial US scanners are widely available for imaging and diagnosis in the clinical setting, to our knowledge, there has never been an approach on using the device for therapy in combination with NBs and genes. In this study, we first fabricated an albumin and lipid-based NBs and evaluated their effect on transfection efficiency *in vitro*. Secondly, a small clinically applicable US imaging device was applied to animals in conjunction with venous injection of genes and NBs. The final goal of the present study was to determine if the US intensities and acoustic parameters from small portable US scanners are adequate as to induce gene transfection in animals.

## Material and Methods

### Preparation of pDNA

pNL1.3CMV [secNluc/CMV] encoding secreted NanoLuc (secNluc) luciferase and pCMV-eGFP encoding the enhanced green fluorescent protein (eGFP) were purchased from Promega (Madison, WI, USA) and Nepagene (Chiba, JP), respectively. The firefly luciferase gene expression vector driven by a cytomegalovirus (CMV) promoter, pCMV-luciferase (Luc), was constructed as previously mentioned (Kawakami et al., 2002).

pDNA was amplified in Escherichia coli strain DH5α. After isolation, pDNA was purified using endotoxin-free plasmid purification kit. The pDNA was dissolved in Milli-Q water and stored at −20 °C prior to each experiment.

### Preparation of Nanobubbles

The solutions of lipid based nanobubbles (L-NBs) and human serum albumin based nanobubbles (A-NBs) were prepared as follows. Firstly, L-NBs were extracted from commercially available US contrast agent, Sonazoid (Daiichi Sankyo, Tokyo, JP), perfluorobutane incapsulated with a phospholipid shell. The powder was dissolved and mixed following the manufacturer's instructions and utilized within 2 h after preparation, however, for *in vitro* experiments, the Sonazoid powder was directly dissolved with revised Eagle’s Minimum Essential Media (opti-MEM: Thermo Fisher Scientific, Waltham, MA USA) instead of distilled water. The bubble including solution was then transferred to the 5 ml tube and centrifuged at 1,200 rpm for 10 min (MX-301; TOMY, Tokyo, JP) to separate the NB and MB component. The necessary amount of NBs within the transparent solution at the bottom of the tube was gently aspirated with a needle (18 G) into a 2.5 ml plastic disposable syringe (Terumo, Tokyo, JP). The 100% solution of NBs was diluted with equal amount of opti-MEM to 50 and 25%. For *in vivo* experiment, Sonazoid powder was reconstituted in 2 ml sterilized distilled water as recommended, and NBs were extracted in the same manner as in the *in vitro* experiments.

Secondly, human serum albumin based NBs were prepared according to a previous study reported elsewhere (Lafond et al., 2018). Briefly, the air in a plastic container tube (height, 30 mm, outer diameter, 25 mm) was replaced with 15 ml of perfluoropropane (C3F8; Takachiho Chemical Industrial, Tokyo, JP) gas using a 23-gauge needle inserted through a small opening in a custom made cap. Ten-ml sterile solution of 0.06% human serum albumin (fraction V, purity 96%; Aventis Behring L.L.C., IL, USA) in opti-MEM (Thermo Fisher Scientific, Waltham, MA, USA) was added in the gas filled container tube. The C3F8 gas and albumin solution in the container were tightly sealed to prevent gas leakage. The container tubes were then placed into a high-speed shaking-type tissue homogenizer device (Precellys Evolution; Bertin Instruments, France) and shaken four times at high speed under the following conditions: 6,500 rpm, 60 s duration, 5 min pause on ice between each shaking phase. After finishing all the shaking phases, the samples were incubated at room temperature for 1 h. To extract uniformly sized NBs from the agitated solution, centrifugation was carried out at 100 g for 10 min to separate all MBs and NBs. Similarly, 0.06% human serum albumin /distilled water NBs were prepared for *in vivo* experiment.

### Characteristic Analysis of Nanobubbles

The physical character of L-NBs and A-NBs were measured as described previously (Watanabe et al., 2019). The particle size of NBs was measured by nanoparticle tracking analysis (NTA) device (NanoSight LM10; Malvern Instruments, Worcestershire, UK). The nanoparticle suspension was illuminated by a 638 nm wavelength red laser. The nanoparticle movement was visualized by light scattering and the Brownian motion recorded by a CCD camera (C11440-50B; Hamamatsu Photonics K.K., Shizuoka, JP). The above system automatically detects the center position of nanoparticles and tracks each particle motion in a two-dimensional plane for later calculation of the average moving distance under Brownian motion. The image of particle movement with NTA was recorded for 60 s at room temperature. The range of particle size measurement of NTA method was adjusted from 10 to 1,000 nm. The particle size was estimated by the average moving distance to the Stokes-Einstein equation. The NBs suspension of 0.5 ml was injected into the sample measurement chamber of the Nanosight system with a 1.0 ml volume plastic syringe (Terumo, Tokyo, JP). Sample image capturing and data analysis were performed using the application software (NTA 3.2 Dev Build 3.2.16). All sample measurement experiments were performed independently for each sample. Particle size was presented as a mean and mode ± standard error of the average of three measurements.

The size and number of L-NBs and A-NBs were measured by a flow cytometer (CytoFLEX; Beckman Coulter, CA, USA). The flow cytometer was equipped with a 405 nm (violet) laser to detect the nanoparticles. The flow cytometer was set up to measure the Side Scatter (SS) from the violet laser for enhanced nanoparticle detection. The Violet-SS signal resolution limitation for particle detection was 200 nm. Superior resolution can be obtained with SS than the Forward Scatter (FS) signal and is suitable for measurement of small particles (e.g. nanoscale particles). In order to relate Violet-SS to a particle size, we calibrated the flow cytometer with beads of known size (Wisgrill et al., 2016; Zucker et al., 2016). The polystyrene standard beads (200, 350, and 800 nm; qNano Calibration Particles; Izon Science, Christchurch, New Zealand, 500 and 1000 nm; Archimedes Standard polystyrene beads; Malvern Instruments, Worcestershire, UK) was suspended in ultrapure water and measured beforehand with the flow cytometer. The acquired Violet-SS signals of lipid and albumin NBs were then analyzed by CytExpert analysis software version 2.0 (Beckman Coulter, CA, USA). A gate was created based on the size of standard beads in the range from 200 to 1,000 nm for determining the size of our fabricated NBs.

### Cell Culture

Oral squamous carcinoma cell line HSC-2 was purchased from JCRB (Japanese Cancer Research Bank) cell bank and cultured in Minimum Essential Medium (MEM; Nacalai Tesque, Kyoto, JP) with 10% Fetal Bovine Serum (In Vitrogen, Tokyo, JP). Cells were maintained at 37.0 °C in humidified air with 5% CO_2_. HSC-2 cells collected by trypsin–EDTA (Gibco, NY, USA). They were then washed and maintained in fresh medium immediately before each sonoporation experiments. On the day before the experiment, cells were collected and centrifuged at 100 g for 5 min. They were seeded by 4.5 x 10^3^/well on 96 multi-well plate dish or Lab-Tek 16 well chamber slide (Thermo Fisher Scientific, Waltham, MA, USA). The cell line was free of viral pathogens with initial viability of more than 99% before use in the actual experiments.

### *In Vitro* Sonoporation Method Using Cell Line on Multi-Wells Plate

**Figure 1:**
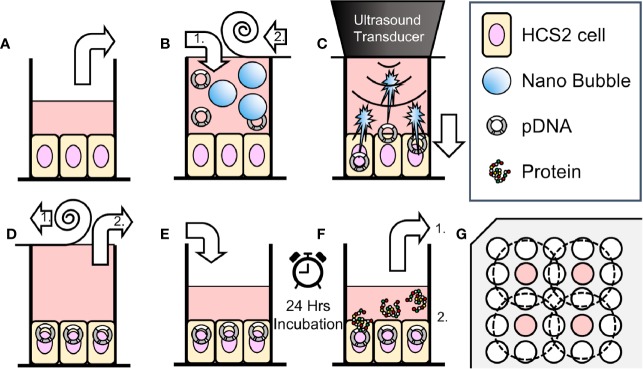
Schematic representation of methods of sonoporation and analysis in 96-well plates. **(A)** Remove incubation medium from well of 96 multi well plate seeded HSC2 cells. **(B)** Fill wells with sonoporation medium, (B-1) and cover well with film (B-2). **(C)** Gene transfection by ultrasonic irradiation. **(D)** Film removal (D-1), and aspiration of battered medium (D-2). **(E)** Add new incubation medium. **(F)** After 24 h incubation, collect supernatant for reporter assay (F-1). Use cells for viability assay (use EGFP plasmid, fix and nuclear staining without collecting supernatant) (F-2). **(G)** Arrangement of wells seeded cells (indicated with color) and ultrasonic irradiation area (inside of dashed circle) on 96 multi-well plates.

pDNA encoding secNluc were respectively attenuated to 10 μg/ml with 25–100% L-NBs or A-NBs and used for *in vitro* sonoporation. The schematic representation of all steps of the experiments are depicted in **Figure 1**.

HSC2 cells were cultured on 96 multi-well plate, every second row and column, in order to prevent interaction of US irradiation to each other (**Figure 1G**). Five types of sonoporation medium; L-NBs of 25, 50, 100%, A-NBs and NBs-free opti-MEM as negative control were prepared for the experiments. Each cell culture wells were replaced and filled with 450 μl in all sonoporation samples pDNA incubated to the upper far edge of the wells followed by sealing the entire plate with acoustically transparent PCR film (Diversified Biotech, Dedham, MA, USA) (**Figures 1A, B**). Great attention was taken to avoid trapping of any gas or air bubbles within the wells. After this procedure, the plate with the PCR seal was placed below the surface of the US transducer via acoustic transmission gel (Aquasonic 100 gel; Parker lab, NJ, USA).

The cells were then exposed to US (**Figure 1C**). The US condition was at the driving frequency of 1 MHz, burst rate of 100 Hz, duty ratio of 50 % and intensity of 5.0 W/cm^2^ and the US was emitted from the transducer (diameter 1.6 cm) (SONIDEL SP100, Sonidel Limited. Dublin, IRE). Each group was sonicated for 5 s in the first series of experiments. In order to clarify the relationship between ultrasonic irradiation time and gene transfer efficiency, the ultrasonic irradiation time was then varied at 0, 5, 10 s in the following experiments. After US irradiation treatment, the wells were emptied and the plate placed upside down on a paper towel to remove excess medium (**Figure 1D**). Then the same volume of medium before irradiation was re-filled to each culture well and incubated for another 24 h (**Figure 1E**). Reporter assay was later performed for measurement of luciferase activity.

eGFP pDNA was diluted to 100 μg/ml of L-NBs in the eGFP activity assay. Each well cultured with HSC2 cells of the 16 well chamber slide was replaced and filled with fresh 450 μl of 100% L-NB sonoporation medium. Then, the wells with secNluc were similarly sonicated (frequency; 1MHz, duty cycle; 50%, burst rate; 100 Hz, intensity; 5.0 W/cm^2^). Sonoporation medium was replaced with new incubation medium in the wells after sonication.

### Animals

Five-week-old male ddY mice were purchased from Kiwa Laboratory Animals (Wakayama, JP) and were housed in a cage in an air-conditioned room and maintained on a standard laboratory diet (CE-2, CLEA, Tokyo, JP) and water. All animal experiments were carried out in accordance with the guidelines for animal experimentation of Nagasaki University.

### *In Vivo* Sonoporation Method

**Figure 2:**
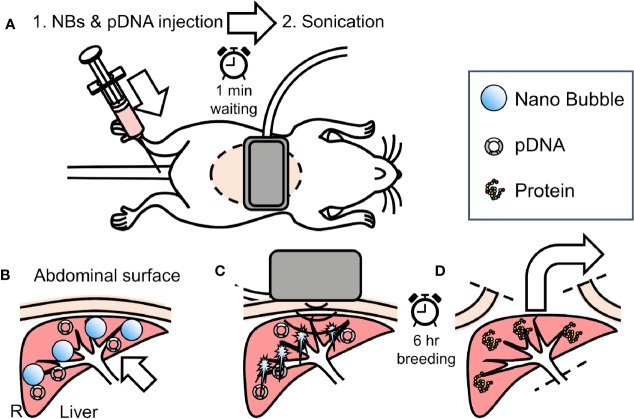
Overview of *in vivo* sonoporation method. **(A)** intraoperative dorsal position. **(B)** accumulation NBs and pDNA into liver from caudal vein through venous and arterial system. **(C)** extracorporeal ultrasonic irradiation using clinical hand-held US imaging scanner. **(D)** 6 h after, sacrifice and harvest of organs for reporter assay.

*In vivo* mice administrated with L-NBs or A-NBs with pDNA encording Luc was sonoporated with a clinical hand-held US imaging scanner under anesthesia. The schematic representation of all steps of the experiments is depicted in **Figure 2**.

Mice were anesthetized using an intraperitoneal injection that contained three types of mixed anesthetic agents (0.5 mg/kg of medetomidine, 4.0 mg/kg of midazolam, and 5.0 mg/kg of butorphanol), hold supine and removed abdominal hair (Kawai et al., 2011). The 300 μl solution consisting of 50 μg/150 μl pDNA in saline and 150 μl L-NBs or A-NBs was bolus injected manually from caudal vein. At 1 min after injection, US was transcutaneously irradiated toward the liver from center of abdomen with a Vscan Dual Probe (GE Healthcare, Waukesha, WI, USA) with Sector Probe (frequency, 2.3 MHz; mechanical index 1.0; thermal index 1.0, depth 6cm, duration 30 s). Mice were freed in the cage after awaking until sacrifice.

### Evaluation of Luciferase Expression

*In vitro* luciferase activity was determined by bis-Coelenterazine(bis-CTZ) assay kit (JNC, Tokyo, JP) using Spark Multimode Microplate Reader (Tecan, Männedorf, Zürich, Switzerland). After 24 h incubation, 10 μl of incubation medium was retrieved on Costar 96-well white solid plate (Corning, NY, USA) each incubation well. Relative luminescence unit (RLU) value was plotted within 10 from 2 s after injection of 1 μg/100 μl bis-CTZ solution. Raw values were corrected with RLU value of blank wells with bis-CTZ solution only.

For evaluation of luciferase in the mice, the luciferase activity was measured as described previously (Fumoto et al., 2016). The liver was excised after 6 h from sonoporation. The extracted liver was homogenized with 5 times the cell lysate. The homogenized sample was centrifuged at 15,000 g for 5 min, at 4°C. Twenty microliters of tissue homogenate supernatant were mixed with 100 μl luciferase assay substrates (PicaGene, Toyo Ink Mfg, Tokyo, JP), The light produced was immediately measured using a luminometer (Lumat LB9507, Berthold Technologies, Bad Wildbad, Germany). Luciferase activity was inversely converted based on the amount of sample measured and the dilution factor with cell lysate, indicated as RLU/g of tissue.

### Evaluation of eGFP Expression *In Vitro*

Cells were sonoporated with eGFP pDNA in 16well chamber slides and were fixed with 4% paraformaldehyde after 24 h of sonication. eGFP was immune-stained with an anti-GFP monoclonal antibody (Invitrogen, Carlsbad, CA, USA) at a dilution of 1:500, an Alexa488-conjugated secondary antibody (Abcam, Cambridge, MA, USA) and Hoechst 33342 (Dojindo Molecular Technologies, Kumamoto, JP) Fluorescence images of cells were analyzed using BZ-X710 digital microscopy (KEYENCE, Osaka, JP).

### Statistical Analysis

Measurement data were displayed as mean ± standard error of the mean (s.e.m). Data was analyzed using unpaired t-test including Welch’s correction. The statistically significant differences between various groups were analyzed using GraphPad Prism 8 software (GraphPad Software, La Jolla, CA, USA) and Microsoft Excel 2016 (Microsoft, Redmond, WA, USA). The probability value of *p* value < 0.05 was considered statistically significant.

## Results

### Characterization of L-NBs and A-NBs

**Figure 3:**
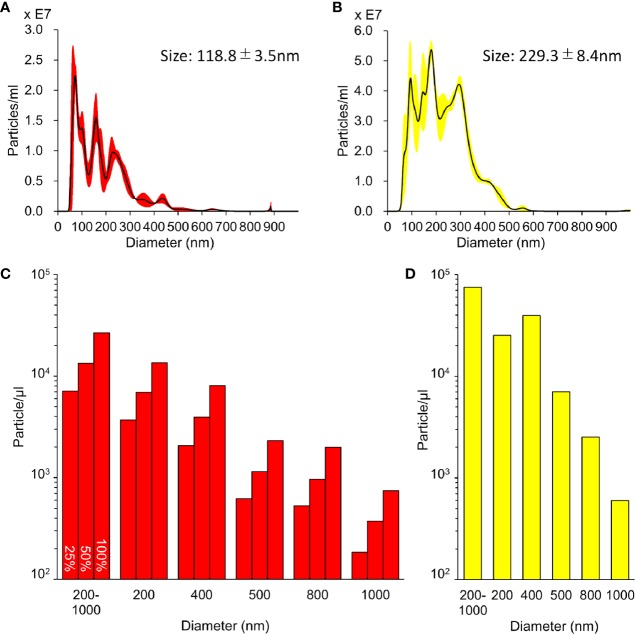
Characterization of sonazoid and albumin NBs. **(A, B)** Detailed distribution and average size of L-NBs **(A)** and A-NBs **(B)** diluted in opti-MEM by NTA. **(C, D)** The concentrations and distribution of L-NBs **(C)** and A-NBs **(D)** based on Violet-SS signal resolution by FCM.

The size distribution data of the L-NBs and A-NBs obtained from NTA is shown in **Figures 3A, B**. The mean size of control sample of L-NBs and A-LB was 118.8±3.5 nm and 229.3±8.4 nm, respectively. The overlaid Violet-SS signal intensity histogram of L-NBs and A-NBs from FCM are shown in **Figures 3C, D**. The number of NBs having the same scattered Violet-SS signal intensity were correlated against standard known particles size ranging from 200 to 1,000 nm. Both 100% L-NBs and A-NBs showed a unimodal distribution with peaks at 200 and 400 nm (modal diameter), respectively. The total number of A-NBs in the 200 to 1,000 nm size range was approximately 7.4 x 10^4^ particles/μl, which was approximately three-fold compared with 0.26 x 10^4^ particles/μl of 100% L-NBs. As expected, the concentration of L-NBs diluted to 25 and 50% was halved step by step while maintaining a 200 nm peak from 100% L-NBs.

### *In Vitro* NBs and US Irradiation pDNA Transfection

**Figure 4:**
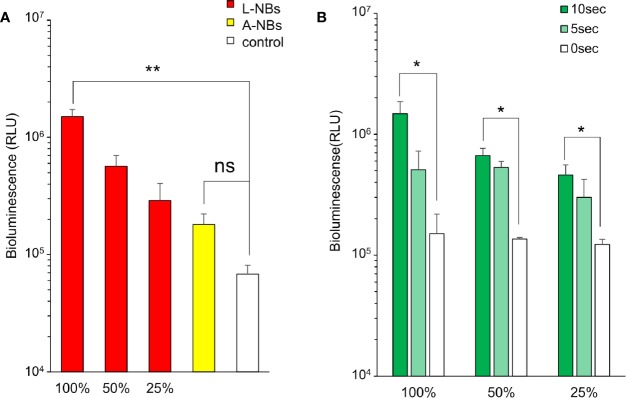
Luciferase expression in HSC2 cells after pDNA administration by sonoporation with NBs. **(A)** Expression of luciferase protein in incubation medium by sonoporation using two types of NBs and concentrations. **(B)** L-NBs concentration and ultrasonic irradiation time dependent profile of luciferase expression. L-NBs, Lipid based nanobubble, A-NB, albumin-based NBs, control, NBs-free opti-MEM including pDNA. RLU, relative luminescence units. The data are presented as the mean ± standard error of the mean (s.e.m.). Statistical significance was assessed by unpaired t-test including Welch's correction. (*p < 0.05, **p < 0.01) (N=3), ns, not significant.

Luciferase-expressing pDNA was introduced into HSC2 cells using various solution with or without NBs and 5 s sonication (**Figure 4A**). 24 h after ultrasonic irradiation, the expression level of luciferase increased in a concentration-dependent manner in L-NBs. The expression level of 100% L-NBs (RLU 15.03 ± 2.76 x10^5^) was significantly increased, which was over 20-fold compared with sonicated opti-MEM without NB with pDNA as negative control (RLU 0.68 ± 0.16 x10^5^). (*p* =0.0065).

Luciferase expression in A-NBs (RLU 1.81 ± 0.51 x10^5^) increase to about three folds compared to negative control but was not statistically significant (*p* =0.1011).

The expression of luciferase was then examined when 25–100% of L-NBs was used by changing the ultrasonic irradiation time to 0 (no irradiation), 5 and 10 s (**Figure 4B**). The amount of expression markedly elevated when the irradiation time was increased to 5 or 10 from 0 s.

**Figure 5:**
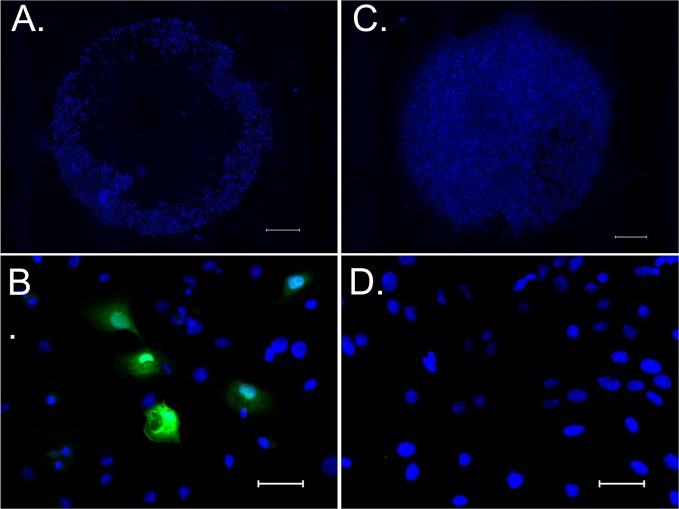
eGFP expression in HSC2 cells after pDNA administration by sonoporation with NBs. **(A, C)** Low power magnified fluorescent microscopic image stained with hoechst 33342 taken 24 h after sonoporation. **(B, D)** High power magnified image stained with hoechst 33342 and anti-GFP antibody. **(A, B)** sonicated sample with pDNA encoding eGFP and L- NBs. **(C, D)** negative control without pDNA, NBs and sonication. Scale bars: 1,000 μm **(A**, **C)**, 50μm **(B**, **D)**.

On immunohistological analysis of HCS2 after introducing pDNA encoding eGFP using 100% L-NBs and 5 s sonication, image of annularly distributed cells on culture well perforated in the center by bubble collapse energy was observed on low-power magnification field (**Figure 5A, C**). Strong eGFP protein expression was detected in cytoplasm on high-power magnification field (**Figure 5B, D**).

### *In Vivo* Sonoporation Using Clinical Hand-Held US Imaging Scanner

**Figure 6:**
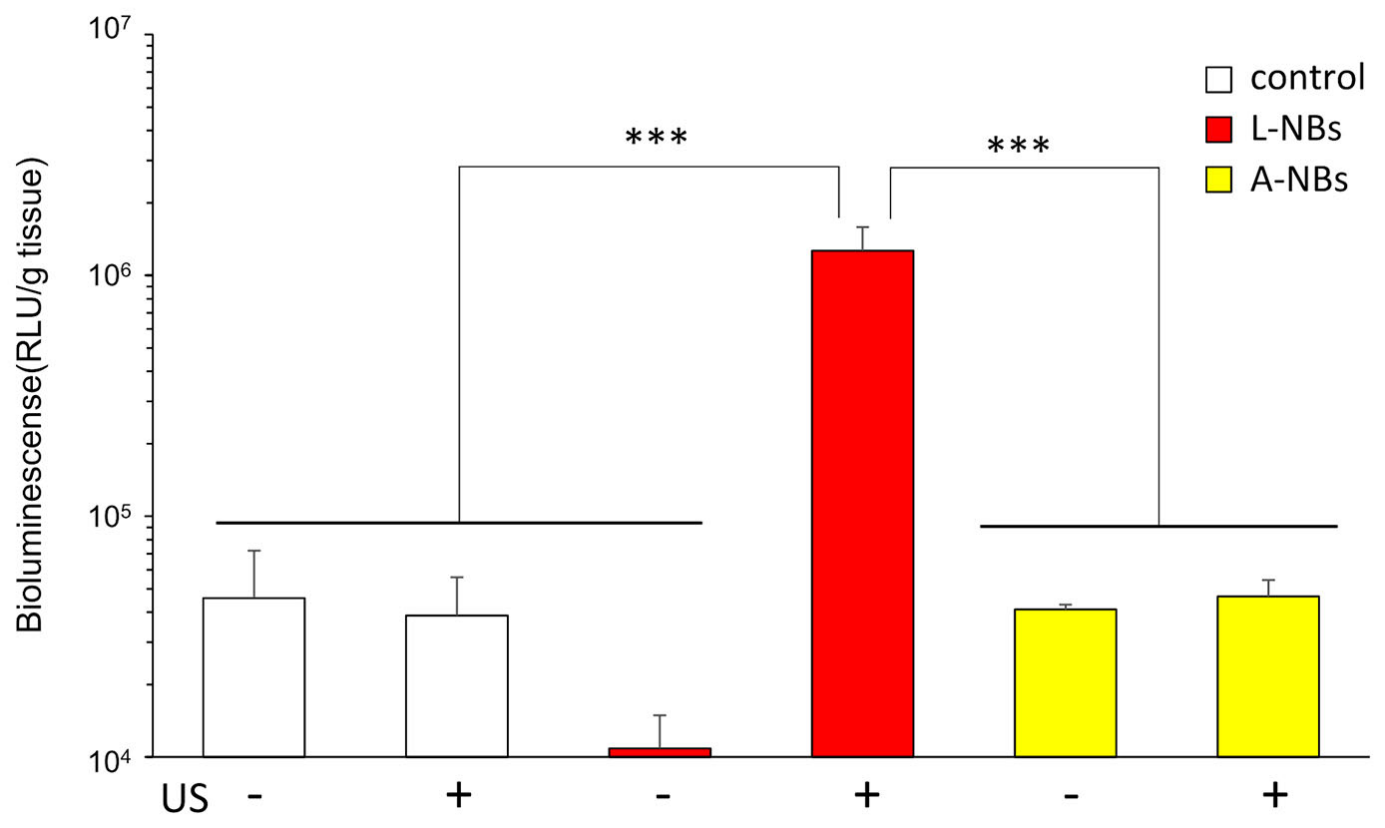
Ultrasound irradiation by clinical hand-held US imaging scanner to the abdomen. Expression of luciferase protein extracted from sacrificed liver after sonoporation using two types of NBs. L-NBs; Lipid-based nanobubbles. A-NB; albumin-based nanobubbles. control, NBs-free saline including pDNA. US, US irradiation. pDNA, plasmid DNA encoding secreted NanoLuc. RLU, relative luminescence units. The data are presented as the mean ± standard error of the mean (s.e.m.). Statistical significance was assessed by Tukey's multiple comparison test. (***p < 0.001) (N=4).

*In vivo* gene transfection at the irradiated liver using L-NBs or A-NBs were evaluated after injection of solution consisting pDNA and NBs from caudal vein and transcutaneous ultrasonic irradiation for liver using a clinical hand-held US imaging scanner (**Figure 6**). In the luciferase activity assay group, a significant increase in expression was detected with the administration of L-NBs and ultrasonic irradiation. The combination of injection of NBs-free solution with pDNA and sonication, or the mixture consisting of pDNA and NBs injection without sonication alone did not result in increased luciferase expression. In the group with solution injected containing A-NBs groups, there was no difference in gene expression with or without ultrasonic irradiation, and was the same as negative control.

## Discussion

In recent years, US-mediated gene therapy has attracted much attention. Various type of MBs and acoustic parameters have been intensively investigated in order to increase gene transfection efficiency. Researchers have added modifications to the bubble shell with marker molecules that bind to targeted tissue sites, for example by coupling specific ligands (Paefgen et al., 2015). The shell can be composed from a variety of materials such as polymers, proteins, phospholipids, and surfactants. We previously compared several commercially available US contrast agents that have different MB shell characteristics and evaluated its gene transfection efficiency in the skeletal muscles (Li et al., 2003). Results showed marked differences in transfection rate depending on shell material. Meanwhile, advancement of technology related to fabrication of smaller sub-micro sized bubbles (Alheshibri et al., 2016) and accurate bubble size measurement modalities (Hernandez et al., 2019) have led to great expectation of its application for gene therapy. Mainly for the reason that sub-micro sized bubbles or nanobubbles may potentially extravasate through the endothelial cell layer of the blood vessel, increased accumulation of NBs in normal tissue and tumor vasculature, can thus be expected. Several researchers have investigated the acoustic character and pharmacokinetics of NBs, suggesting possible involvement of EPR effect (Tong et al., 2013; Wu et al., 2013; Cavalli et al., 2015; Du et al., 2018). Furthermore, it has recently been reported that the time-intensity-curve of extravasated NBs can be detected in tumors (Wu et al., 2019). Histologic analysis showed that NBs were retained in tumor tissue to a greater extent compared with MBs.

Lafond et al. (2018) recently demonstrated that their in-house fabricated albumin-based NBs, which is 100 to 250 nm in diameter were sensitive to specific acoustic pressures and could become efficient cavitation nuclei in the 3 to 5-MHz US frequency range. Acoustic passive cavitation detectors indicated that inertial cavitation threshold was lower than the commercially available MB US contrast agents. Watanabe et al. (2019) similarly conducted a detailed study of albumin-based NBs by 3 different nanoscale measurement modalities and characterized the bubble size distribution, gas/particle ratio, and concentration. Subsequently, superior contrast imaging and elevation of time signal intensity curve of the NBs were obtained in an *in vitro* flow vessel system driven at US frequencies of 40 MHz. They later went on to irradiate US to cultured cancer cells with therapeutic US (1 MHz) in the presence of NBs that functioned as cavitation nuclei thus potentiating acute cell disruption. In the present study, similar US conditions were irradiated to *in vitro* cultured cancer cells which resulted in induction of gene transfer. It is postulated that the NBs became cavitation nuclei to induce sonoporation at the cellular level, thus resulting in enhanced gene transfer. However, it is not clearly understood why lipid-based NBs resulted in significantly higher transfection rate compared to albumin-based NBs, in spite of the fact that both had similar size distribution and concentration. As this tendency was observed both in our *in vitro* and *in vivo* experiments, it is suggested that NB shell material may have attributed to this phenomenon but further verification is needed, as well as, considering the influence of bubble diameter which cannot be completely excluded from an acoustic stand point.

Nanobubbles have previously been applied for US mediated gene delivery in different anatomical locations. Nishimura et al. (2019) reported site-specific transgene expression locally at the defined area of the peritoneum with NB induced sonoporation. Naked pDNA and NBs were directly administered *in vivo* and multi-color deep imaging analysis revealed that the transgene expression can be in the peritoneal mesothelial cells. It was suggested that intraperitoneal gene delivery by sonoporation might be an effective therapeutic method for treatment of peritoneal fibrosis. Alternative drug delivery strategies have been reported by fabrication of US-sensitive siRNA-loaded polymeric micelles and liposome NBs for gliomas (Yin et al., 2013). Relatively low-frequency US was irradiation to induce release of siRNA micelles in tumor tissue from the siRNA loaded NBs and then effectively delivered into cancer cells. Furthermore, several promising data have been reported on liver gene therapy by combining various types of NBs and US (Wu et al., 2016; Zhang et al., 2018). Nevertheless, our animal sonoporation study also revealed induction of gene transfer in the liver after intravenous injection of lipid-based NBs. Our preliminary US irradiation (frequency 1.0 MHz, intensity 1.0 W/cm^2^) experiments (**Supplementary Figure S1**) showed that NB/gene administrated mice had higher transfection rate in the liver compared to the spleen and kidney, however, the exact mechanism for these results is unclear. It is speculated that the acoustic parameters, especially the US intensity may greatly alter the rate of gene transfer. Dimcevski et al. (2016) conducted a human clinical trial using diagnostic US device and MBs to enhance gemcitabine treatment of inoperable pancreatic cancer. In their study, the echography scanner configuration was programmed to maximize the duty cycle, with short broadband linear pulse in order to excite as many MBs as possible. In our study, although US parameters were preset to supposedly maximum intensity, it was anticipated that sufficient acoustic peak pressure may not reach the target organ using such small low powered hand-held US scanners. Fortunately, gene transfer was detected at least in the most proximally located organ to the US scanner probe of this particular FDA approved device. In this respect, the present study provides limited information on the optimal acoustic conditions for NB induced sonoporation. It is speculated that a more acoustically customized US scanner can further improve the efficiency of gene transfer. It would thus be worthy to evaluate various parameters as well as the NB shell materials for future gene therapy experiments.

## Conclusion

Ultrasound-mediated sonoporation induced gene transfection in both *in vitro* and *in vivo* in the presence of lipid-based NBs. Furthermore, we demonstrated that commercially available hand-held US scanners have sufficient acoustic pressure as to induce gene transfection in animal liver. Additional acoustic parameter studies should be carried out to investigate the applicability of this modality for gene therapy.

## Data Availability Statement

The raw data supporting the conclusions of this article will be made available by the authors, without undue reservation, to any qualified researcher.

## Ethics Statement

The animal study was reviewed and approved by Regulations of the Animal Care and Use Committee, Nagasaki University.

## Author Contributions

KT, HK, LF, YI, and HE were involved in the design, analysis, data collection, and preparation of the manuscript. KN, KO, AW, and SK carried out the animal experiment, data analyses, and interpretation of the results. KT, HK, and AW contributed to the writing of the manuscript and revision of the text.

## Conflict of Interest

KT owns stock in SonoCore Inc.

The remaining authors declare that the research was conducted in the absence of any commercial or financial relationships that could be construed as a potential conflict of interest.
