# Supplementary material for: Nanobubble Mediated Gene Delivery in Conjunction With a Hand-Held Ultrasound Scanner
Source: Front Pharmacol. 2020 Apr 1;11:363. doi: 10.3389/fphar.2020.00363 (PMC7145407; doi:10.3389/fphar.2020.00363)
Supplement: Supplementary file 1 [file Image_1.pdf]

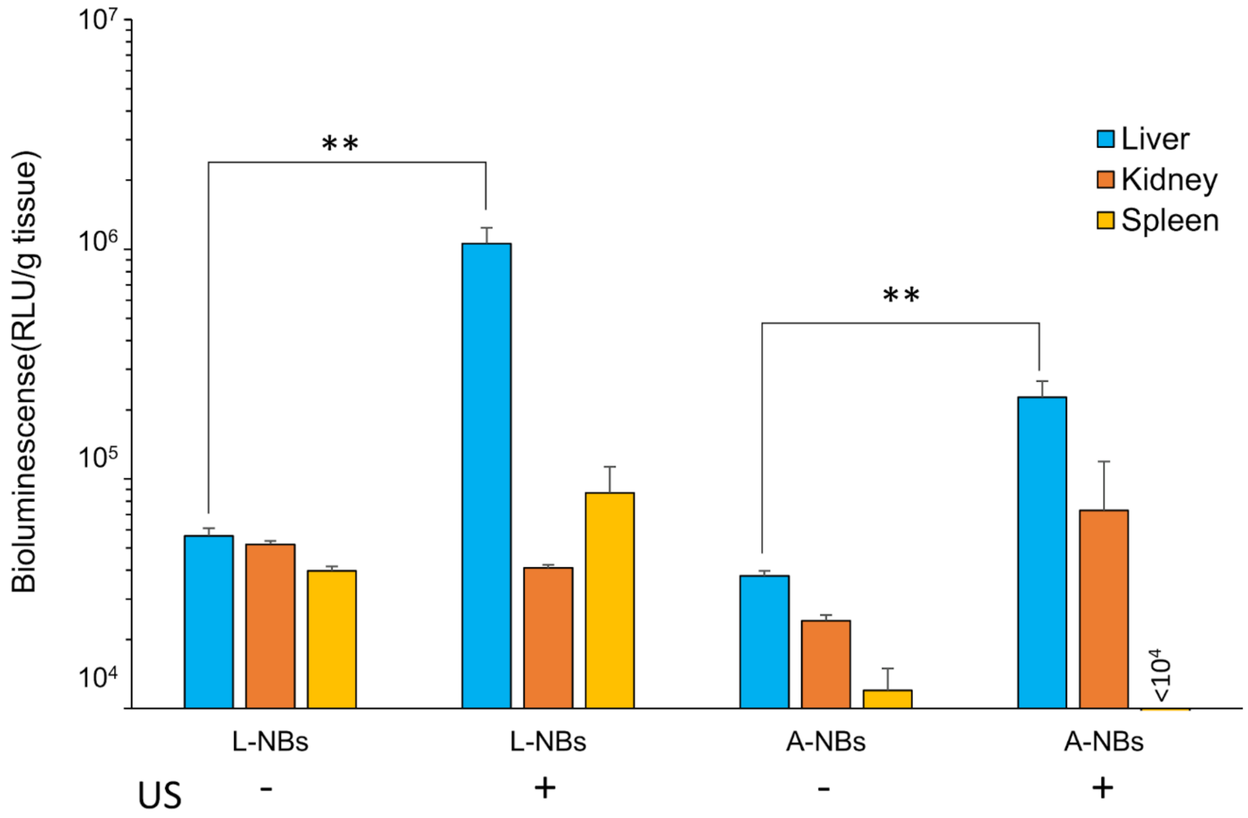

Figure S1. Luciferase activity (RLU/g tissue) was measured different organ tissues in mice, 6hrs after intravenous injection of gene and ultrasound irradiation (KTAC-4000, NepaGene, Chiba, JP). Ultrasound frequency: 1.0MHz, Intensity 1.0W/cm2, Duty ratio 50%, Pulse repetition frequency 10Hz, irradiation time 30sec. Data was analyzed using unpaired t-test including Welch's correction. A probability value of $P<0.05$ was considered statistically significant. (n=4)
